# A survey of NHS nurses' delivery of treatments to prevent recurrence of venous leg ulcers

**DOI:** 10.1111/iwj.70101

**Published:** 2025-01-12

**Authors:** Abeer Muflih Alkahtani, Jo C. Dumville, Christopher J. Armitage

**Affiliations:** ^1^ Division of Nursing, Midwifery and Social Work School of Health Sciences, University of Manchester Manchester UK; ^2^ Division of Psychology and Mental Health School of Health Sciences, University of Manchester Manchester UK; ^3^ Manchester Academic Health Science Centre, Manchester University NHS Foundation Trust Manchester UK; ^4^ NIHR Greater Manchester Patient Safety Translational Research Centre, University of Manchester Manchester UK

**Keywords:** COM‐B, endo‐venous ablation surgery, NHS nurses, recurrence of venous leg ulcers, strongest tolerated compression

## Abstract

Preventing recurrence of venous leg ulcers can be achieved through strongest tolerated compression and endo‐venous ablation surgery, but it is not clear how often this is done in practice. This study explores (1) nurses' awareness of strongest tolerated compression and endo‐venous ablation surgery as prophylactic treatments for venous leg ulcer, (2) how often these treatments are offered, and (3) assessment of the barriers and enablers to deploying those treatments using the capabilities, opportunities and motivations model of behaviour change. An online cross‐sectional survey was conducted among nurses who treat and manage venous leg ulcers across the United Kingdom. Data were analysed descriptively using within‐participants ANOVA, within‐participants MANOVA and multiple linear regression. We received 96 questionnaire responses. All the respondents reported that they were aware of strongest compression to prevent recurrence while 87.5% reported they were aware of endo‐venous ablation surgery for recurrence prevention. Nurses' capabilities, opportunities, and motivations to offer the strongest tolerated compression were significantly greater when offering the strongest compression compared with referring to vascular surgery. Both preventative treatments were associated with marked deficits in opportunities (social and physical) and automatic motivation. Interventions targeted at increasing nurses' opportunities and boosting their motivation are needed to support the delivery of both preventive treatments. Further research is required to gain in‐depth understanding of those barriers and enablers to identify candidate behaviour change techniques.

## 1 BACKGROUND

Venous leg ulcers (VLUs) are common, recurring open wounds on the lower leg, between the ankle and the knee, which occur due to impaired blood flow through damaged or diseased leg veins, leading to the breakdown of the skin and poor healing. VLUs are associated with pain, impairment of mobility and negatively impact the quality of life of the people with VLUs.^1^, ^2^ Internationally, a recent meta‐analysis included thirteen studies from twenty‐six countries, reported a pooled prevalence of 0.32% (95% confidence interval (CI) 0.129%–0.595%).^3^ Two community‐based multiservice epidemiological studies conducted in the United Kingdom (UK) estimated the point prevalence of VLUs at approximately 0.29 cases per 1000 population in Leeds (95% CI: 0.25–0.33),^4^ and 3.2 cases per 10 000 population in north England (95% CI: 2.9–3.4).^5^ The estimated annual cost to the UK National Health Service (NHS) of treating people with a VLU was £102 million or, on average, £4787.70 per patient.^6^

VLUs are associated with cycles of ulceration, healing and recurrence. As these wounds are symptoms of an underlying venous problem, they often recur following healing. An ulcer is considered recurrent if there is a repeat event anywhere on the treated leg.^7^ Evidence on the incidence of VLU recurrence is relatively scarce and varied.^8^ In a UK trial of compression treatments, of the 343 participants classed as having an ulcer free leg during the study, 65 (19%) had a recurrence within 12 months.^9^ An earlier compression trial reported recurrence following healing by arm only, with 36.2% and 39.1% of participants having a recurrence.^10^ Secondary analyses of combined data from three longitudinal studies assessed ulcer recurrence rates in Australia (total *n* = 250; median follow‐up 17 months). Six months following ulcer healing, 39% of participants reported an ulcer recurrence, increasing to 57% at 12 months. The median time to recurrence was reported as 42 weeks.^11^

Compression therapy and endo‐venous ablation surgery are evidence‐based preventive treatments for VLU recurrence. Compression therapy improves blood flow in the leg or legs via the application of tight, often multi‐layered bandages or stockings.^12^ The pressure applied by these devices alleviates venous insufficiency, in turn, promoting healing and preventing recurrence.

Following VLU healing, UK Guidelines recommend wearing below‐knee graduated compression hosiery/stockings that apply a strong graduated compression (at least 40 mmHg) to prevent recurrences of VLUs.^12^ A recent Cochrane review^7^ that included eight studies with 1995 participants, supports these recommendations, finding that European (EU) class 3 compression stockings (delivering between 37 and 47 mmHg of compression) compression tighter (that is higher) compression offers better outcomes. Endo‐venous ablation, where saphenous vein ablation is also used to eliminate underlying venous incompetence in legs with isolated superficial venous reflux, has been shown to reduce VLU recurrences^13^, ^14^, ^15^ and is recommended by UK guidelines.^12^

In the UK, most VLU care is delivered by nurses in community settings, with the oversight of tissue viability specialist nurses (TVNs). Nurses are normally trained in the delivery of compression; however, the availability of referral pathways for nurses to facilitate access for patients to surgery is not clear. Despite robust evidence for preventive options, VLU recurrence is an on‐going issue suggesting that those preventive treatments are not being delivered as optimally as they could be.

Previous research has focused primarily on exploring the challenges nurses encounter when delivering compression as a therapy for active VLUs, where healing is the outcome of focus.^16^, ^17^, ^18^, ^19^ However, behaviours around the delivery of, and possible enablers and barriers to post‐healing care by nurses to prevent recurrence, has not been investigated. Consequently, there are crucial gaps in understanding nurses' awareness and delivery of two key VLU prophylaxis: strongest tolerated compression stockings/hosiery and referral of people with a history of VLU to vascular services to be considered for endo‐venous ablation surgery. We also need insights into barriers and enablers for both preventive treatments from nurses' perspectives utilising a validated behaviour change framework.

### 1.1 Theoretical framework

The capabilities, opportunities and motivations model of behaviour change (COM‐B) is endorsed by the UK National Institute for Health and Care Excellence as a key theoretical framework for understanding and supporting behaviour change.^20^ COM‐B comprises six components: Capability which is defined as an individual's psychological (e.g., knowledge) and physical (e.g., skill) capacity to engage in the behaviour concerned; Opportunity which is defined as all the factors that lie outside an individual that make behaviour possible or prompt it, which can be physical (e.g., time) or social (e.g., social cues); and Motivation which is defined as all the brain processes that energise and direct behaviour (B), which can be reflective (e.g., evaluations and plans) or automatic (e.g., emotions and impulses).^21^, ^22^, ^23^ COM‐B has been used to identify barriers and enablers in different areas of healthcare^24^, ^25^, ^26^ but not yet to understanding the barriers to and enablers of nurses' delivery of preventative treatments for VLU recurrence.

The present study aims to assess for the first time (1) whether NHS nurses are aware of strongest tolerated compression hosiery and endo‐venous ablation surgery as evidence‐based preventative approaches, (2) nurses' perceptions of how many of their patients would benefit from these approaches and how frequently they offer these to patients and (3) the barriers to and enablers of offering these preventative approaches.

## 2 METHODS

### 2.1 Study design and respondents

A cross‐sectional survey design was employed. All NHS nurses across the UK who treat patients with VLUs were eligible to participate. This sampling frame aimed to gather a wide range of experiences with as much variation as possible among the respondents regarding awareness, provision and their capabilities, opportunities and motivations for offering these preventive treatments.

The respondents were purposively sampled. The study was reviewed and exempted from ethical approval by the University of Manchester Ethical Committee due to anonymised data collection and the nature of questionnaire items, which were strictly limited to the respondents' professional competence and not asking personal or sensitive questions (letter of exemption available on request). Informed consent was obtained from the respondents prior to the completion of the questionnaire.

### 2.2 Instrument

#### 2.2.1 Development and measures

To explore the challenges encountered while providing treatments for the prevention of VLU recurrence, an online survey was constructed and administered utilising Qualtrics platform.^27^ The questionnaire included three sections: (1) demographic data related to gender, age, ethnicity, experience, band and the NHS region of work in the UK; and (2) items related to offering the strongest tolerated compression and referring patients to vascular services for endo‐venous ablation surgery. The respondents were asked about their awareness of these interventions for preventing VLU recurrence. They were also asked to rate (using a 0%–100% rating scale): (a) what proportion of people with healed VLUs they see in a typical working month they thought would benefit from using strongest tolerated compression and referral to vascular services and (b) of the patients they felt would benefit, what proportion they actually offered these treatments to. The last section (3) explored nurses' self‐reported capabilities, opportunities and motivations with regard to offering strongest tolerated compression and referring patients with VLUs to vascular services for consideration for surgery. This final part of the questionnaire was informed by the COM‐B model.^21^ Tables 1, 2, 3 illustrate the survey items.

**TABLE 1:** Sample characteristics (*n* = 96).

Variable	*n*	%	Mean %	SD
Gender				
Male	5	5.1		
Female	90	93.8		
Not stated	1	1.0		
Age, years			46	10.8
Ethnicity				
White	82	85.4		
Other ethnic group	14	10.4		
Nursing role				
Community nurse	23	24.0		
Tissue viability nurse	47	49.0		
Other nursing role	27	27.1		
Experience, years			17.09	12.5

**TABLE 2:** Awareness and prevalence of venous leg ulcer recurrence preventive treatments (*n* = 96).

Variable	Compression				Surgery			
	*n*	%	*M*	SD	*n*	%	*M*	SD
(Awareness)								
Before today, were you aware of ‘the strongest level of compression hosiery tolerated’ as a treatment for people with healed venous leg ulceration to prevent recurrence?								
Yes	96	100%			84	87.5%		
No	0				12	12.5		
(Provision)								
When you offer compression hosiery to people with healed venous leg ulcers, what is the strongest level of compression you normally aim for?								
Class 1 (14–17 mmHg) light	5	5.2						
Class 2 (18–24 mmHg) Medium	46	47.9						
Class 3 (25–40 mmHg) Strong support	38	39.6						
I do not offer compression hosiery to this patient group	7	7.3						
(Prevalence)								
Of the people with healed venous leg ulcers you see in a typical working month, what proportion do you think would benefit from having the strongest level of compression hosiery they can tolerate to prevent recurrence?			83.6	23.6			55.9	26.8
Of people with healed venous leg ulcers you see in a typical working month who you think would benefit, with what proportion do you offer of the strongest level of compression hosiery tolerated to prevent recurrence?			67.9	29.4			37	30.8

**TABLE 3:** Capabilities, Opportunities and Motivations to offer Strongest tolerated compression and referring to surgery (*n* = 96).

Variable	Offering compression		Referring to surgery		df	*F*	*p*	Partial eta squared
	*M*	SD	*M*	SD				
Physical opportunity	6.57	2.95	4.9	3.1	1,190	13.588	<0.001	0.067
Social opportunity	6.16	2.9	4.9	3.0	1,190	8.286	0.004	0.042
Reflective motivation	8.39	2.16	7.3	3.33	1,190	7.258	0.008	0.037
Automatic motivation	7.8	3.33	5.4	3.46	1,190	28.508	<0.001	0.130
Physical capability	8.54	2.15	7.17	3.46	1,190	10.763	<0.001	0.054
Psychological capability	8.9	1.42	8.02	2.96	1,190	7.237	0.008	0.037

The survey items to assess nurses' awareness of VLUs recurrence preventative treatments and the perceptions of those preventative treatments were adapted from Keyworth et al.^28^ A previously validated tool was utilised to collect data on nurses' self‐reported capabilities, opportunities and motivations (Appendix 1).^29^ The measure comprises six items to assess respondents' capabilities, opportunities and motivations. Brief definitions of each construct are given for respondents, following the questions being posed, where wording was tailored to the clinical focus of this study. Response options were captured on an 11‐point scale: strongly disagree [0] to strongly agree [10].

It is important to pilot questions and modify their content based on this process.^30^ The survey was piloted with five clinical and research experts. This stage aimed to identify any problems with question phrasing, comprehension, instrument length, format or any other element. The survey was subsequently modified based on the pilot findings.

#### 2.2.2 Survey distribution

The responses were collected over 4 months (February 2023–June 2023). The survey was circulated via health professional organisations such as the Tissue Viability Network, Wounds Research Network, National Wound Care Strategy Programme and Queen's Nursing Institute. Each professional organisation's administrator has been contacted and asked to share the survey with their nurses. The survey is detailed in Appendix 2.

#### 2.2.3 Statistical analysis

A six‐step approach to multivariate analysis^31^ was followed. The flowchart of the steps followed can be found in Appendix 3. Statistical Package for the Social Sciences version SPSS 28.0.1.0^32^ was used to analyse the data. Descriptive analysis was employed, using simple summary statistics, percentages for categorical data and mean values with standard deviations for numerical data, to quantify prevention‐related activities. Within‐participants analysis of variance (ANOVA) was used to contrast the differences between levels of capabilities, opportunities and motivations with respect to each preventative treatment. Within‐participants multivariate ANOVA (MANOVA) was used to compare levels of capabilities, opportunities and motivations between behaviours. Finally, multiple linear regression was employed to examine the association between levels of capabilities, opportunities and motivations and behaviours for each preventative treatment. The Checklist for Reporting Results of Internet E‐Surveys (CHERRIES)^33^ was followed when reporting the survey (Appendix 4).

#### 2.2.4 Dealing with missing data

A four‐step process for identifying missing data and applying remedies was utilised.^34^ The process followed can be found in the Appendix 5. Missing data patterns were observed and tested using SPSS 28.0.1.0. The observation and testing of missing data indicated that data were missing completely at random.^35^ Multiple imputations were applied to generate multiple datasets and provide accurate estimates of the uncertainty associated with missing data.^34^, ^35^ Finally, the pooled data were tested using a T‐test bootstrap to compare the multiple imputed data with missing values. This was done to confirm that there was no statistically significant difference between the two sets of data and to account for uncertainty resulting from sampling fluctuations.^31^, ^36^

## 3 RESULTS

### 3.1 Sample characteristics

The online survey was opened by 123 nurses, but 96 were partially or completely completed. In total, 96 questionnaire responses were received and analysed. Most of the respondents were white (*n* = 82, 85.4%), and the majority were female (*n* = 90, 93.8%). The mean age was 46 years (SD = 10.8) and the mean number of years of experience of delivering wound care was 17.2 (SD = 12.4). The sample included tissue viability nurses (*n* = 47, 49.0%), community nurses (*n* = 23, 24.0%), practice nurses (*n* = 7, 7.3%) and other nursing roles (*n* = 19, 19.8%). Most respondents provided VLU care in the patient's place of residence (*n* = 44, 28.8%), a community clinic/treatment room (*n* = 36, 23.5%) and in‐patient settings (*n* = 26, 16.9%). Table 1 displays an overview of the sample characteristics.

### 3.2 Nurses' awareness of treatments to prevent VLU recurrence

All the respondents (*n* = 96, 100%) reported that they were aware of the strongest tolerated level of compression as a preventative treatment. In contrast, their awareness of endo‐venous ablation surgery was statistically significantly lower (*n* = 84, 87.5%), (95% CI: −0.19236 to −0.57641924, *p* < 0.001) (Table 2).

### 3.3 Behaviours (B)

In a typical working month, nurses perceived that more people would benefit from offering strongest tolerated compression than from referral to vascular surgery (83.7% compared with 55.9%). Nurses, then reported that, on average, they recommended strongest tolerated compression hosiery to 69.7% of people they thought would benefit and just 37.0% of people they thought could benefit from referral to vascular services (Table 2).

The strength of compression hosiery offered varied with almost half of respondents (47.9%) reporting they aimed for UK Class 2 (18–24 mmHg) medium support, and 39.6% reported aiming for Class 3 (25–40 mmHg) strong support hosiery and 5.2% of respondents reported aiming for Class 1 (14–17 mmHg). Finally 7.3% of the respondents reported they did not recommend compression.

### 3.4 Capabilities, Opportunities and Motivations for the delivery of recurrence of VLUs preventative treatments

Respondents scored highest in relation to their capabilities and reflective motivation to deliver both preventative treatments. However, they scored lower when considering referral to vascular services compared with offering strongest tolerated compression hosiery. Considering capabilities, respondents mean psychological capability scores were 8.9 (SD = 1.42) when asked about offering strongest tolerated compression and *M* = 8.02 (SD = 2.96) when asked about referral to vascular services. Their physical capability was also higher for offering strongest tolerated compression *M* = 8.54 (SD = 2.15), compared with referral to vascular services *M* = 7.17 (SD = 3.46). Considering reflective motivation, respondents again scored higher when asked about offering strongest tolerated compression hosiery *M* = 8.39 (SD = 2.16) compared with referral to vascular services *M* = 7.3 (SD = 3.33). The mean score for automatic motivation was 7.8 (SD = 3.33) for offering strongest tolerated compression hosiery and lower for referral to vascular services *M* = 5.4 (SD = 3.49), meaning on average respondents felt they had the required skills, stamina and desire to offer both preventative treatments to people with a history of VLUs to prevent recurrence. Physical and social opportunities to deliver both preventative treatments were the lowest scoring constructs. Physical and social opportunity mean scores were 6.57 (SD = 2.95) and 6.16 (SD = 2.9) respectively, for offering strongest tolerated compression compared with means of 4.9 (SD = 3.1) and 4.9 (SD = 3.0) for referral to vascular services on a scale of 0–10 (Table 3).

### 3.5 Differences in nurses' Capabilities, Opportunities and Motivations between both preventive treatments

Within persons MANOVA revealed that nurses' perceptions of their capabilities, opportunities and motivations to refer VLU patients to vascular services for recurrence prevention were significantly lower than their perceptions of their capabilities, opportunities and motivations to provide the strongest tolerated compression. There was a significant statistical difference in respect to COM for both behaviours (Table 3).

### 3.6 Zero‐order correlations between behaviours and COM

Table 4 presents the zero‐order correlations between both behaviours and COM. The correlations between the delivery of preventative treatments and COM components are statistically significant, ranging from *r* = 0.29–0.64, indicating ‘medium‐sized’ effects.^37^ A large correlation exists between automatic motivation (*r* = 0.64) and referral to vascular surgery which *r* exceed *r* = 0.50.

**TABLE 4:** Zero‐order correlations among capability, opportunity and motivation components and behaviours (*n* = 96).

Variable	Physical opportunity	Social opportunity	Reflective motivation	Automatic motivation	Physical capability	Psychological capability
Offering strongest compression	0.32	0.34	0.29	0.39	‐	‐
Physical opportunity	‐	0.63	0.54	0.39	0.41	0.29
Social opportunity	.‐	‐	0.55	0.44	0.38	0.45
Reflective motivation	‐	‐	‐	0.35	0.52	0.38
Automatic motivation	‐	‐	‐	‐	0.52	0.30
Physical capability	‐	‐	‐	‐	‐	0.49
Referring to vascular surgery	0.41	0.45	0.32	0.64	0.41	0.43
Physical opportunity	‐	0.76	0.35	0.40	0.28	‐
Social opportunity	‐	‐	0.44	0.42	0.38	0.28
Reflective motivation	‐	‐	‐	0.23	0.28	0.22
Automatic motivation	‐	‐	‐	‐	0.57	0.52
Physical capability	‐	‐	‐	‐	‐	0.57

### 3.7 Association between Capability, Opportunity and Motivation of both preventative treatments

Multiple linear regression (Table 5) showed that nurses' perceptions of their automatic motivation and their opportunities (physical and social) were most strongly associated with optimal delivery of VLUs recurrence preventative treatments. The *R* ^2^ for offering strongest compression and referring to vascular services was = 0.256 and 0.476, respectively.

**TABLE 5:** Association between capability, opportunity and motivation and offering strongest tolerated compression and referring to vascular surgery (*n* = 96).

Variable	Compression				Surgery			
	*B*	SE	95% CI	*p*	*B*	SE	95% CI	*p*
Physical capability	0.479	1.409	−2319, 3277	0.735	2.225	0.985	0.268, 4.182	0.026
Psychological capability	2.239	2.114	−1.959, 6.439	0.292	3.029	1.162	0.721, 5.337	0.011
Physical opportunity	3.190	0.971	1.262, 53 119	0.001	1.533	1.374	−1.194, 4.261	. <0.001
Social opportunity	3.406	0.984	1.452, 5.359	<0.001	3.376	1.453	0.941, 6.261	0.022
Motivation	3.090	1.362	0.356, 5.794	0.026	1.696	0.735	0.237, 3.155	0.023
Automatic motivation	4.096	0.997	2.116, 6.076	<0.001	5.347	0.707	3.942, 6.752	<0.001

Abbreviation: CI, confidence interval.

## 4 DISCUSSION

This is the first study in the UK to assess (1) NHS nurses' awareness and delivery of evidence‐based preventive treatments for VLU recurrence and (2) nurses' perceptions of their capabilities, opportunities and motivations for preventing VLU recurrences are explored using a validated COM‐B measure.

The survey was distributed to nurses who treat VLUs across the UK. We found that respondents were aware of the need to offer the strongest tolerated compression hosiery, although around 16% of respondents reported not perceiving that patients would benefit from this. Respondents reported recommending strongest tolerated compression to around 70% of those they thought would benefit.

Respondents were less aware of endo‐venous ablation surgery as a preventative treatment compared with strongest tolerated compression. Furthermore, only 55.9% of respondents thought their patients would benefit from referral to vascular services and, of these, only 37% reported referring their patients. So, as well as awareness there may be other barriers to making referrals.

In general, nurses' capabilities, opportunities and motivations to offer strong tolerated compression were greater than their capabilities, opportunities and motivations to refer people with healed VLUs to vascular services. Nurses' self‐reported opportunities scored relatively low for both preventive treatments. Previous work suggests that limited time and resources (physical opportunities) due to workload and staffing issues are on‐going issues for nurses delivering care.^18^, ^19^, ^38^, ^39^ Providing time and resources (physical opportunities) and creating social norms to enhance social opportunity^21^ are prime targets for intervention.

When considering the use of compression as a treatment for VLUs, the provision of training to mitigate gaps in practitioner knowledge and skills around compression use have also been identified in previous work.^18^ These may be an issue for prevention activities too, although there has been less exploration of this. Previous work has also highlighted the importance of patient knowledge about why compression is needed to support on‐going use of this treatment. There has been less exploration of this in relation to the provision of surgery for this patient group, but collective insights into patient's understanding of their venous damage or disease that underpins their on‐going risk for venous leg ulceration would be interesting to further explore. Better understanding here may offer insights in whether the opportunities nurses have to work with patients to support VLU prevention can be enhanced.

It is also important to consider the differences observed between compression and referral to specialist vascular services. Offering compression therapy can be considered a core activity for nurses delivering VLU care. However, when previous research explored how referrals to vascular specialists are made, the majority of respondents noted that GPs needed to make these referrals.^16^ There may be barriers to suitable pathways that give nurses the opportunities to make timely and appropriate referrals, preventing referral from becoming embedded into their practice and routine activities, perhaps explaining the lower automatic motivation score; this requires further exploration.

### 4.1 Strengths and limitation

This is the first study to examine NHS nurses' awareness and delivery of strongest tolerated compression and referral to vascular surgery to prevent VLU recurrence. The study is also the first study to assess nurses' capabilities, opportunities and motivations to apply those preventive treatments using a validated tool which has been beneficial in predicting behaviour and identifying barriers and enablers to various targeted behaviours.^40^, ^41^

This study has some limitations. Whilst the study aimed to reach nurses who treat VLUs across the UK, we received a relatively limited number of responses. In addition, despite survey pretesting, missing data rates were high. Additionally, since the survey was completely anonymised, it was not possible to prevent multiple entries from the same respondent. A further limitation with cross‐sectional surveys design is that the data rarely probe deeply into the meaning of phenomena to the respondents and do not allow causal inference.^42^ However, we are undertaking a qualitative study to gain an in‐depth understanding of barriers and enable both preventative treatments.

## 5 CONCLUSIONS

Understanding nurses' capabilities, opportunities and motivations to offer strongest compression and refer people with a history of VLU to vascular surgery to prevent the recurrence is crucial to enhance the delivery of VLU recurrence evidence‐based preventive treatments. Although this survey provides some insight into VLU recurrence prevention, it only provides an overview of the delivery of those preventive treatments. Hence, more detailed and in‐depth research using, for example, the Theoretical Domains Framework (TDF) would be useful for understanding the drivers underpinning nurses' influences of optimal care delivery post‐healing. The TDF is a comprehensive theory‐informed approach to identify determinants of professional behaviours^43^.

## CONFLICT OF INTEREST STATEMENT

The authors declare no conflicts of interest.

## Supporting information


**Data S1.** Supporting information.

**Data S2.** Supporting information.

**Data S3.** Supporting information.

**Data S4.** Supporting information.

**Data S5.** Supporting information.

## Data Availability

The data that support the findings of this study are available from the corresponding author upon reasonable request.
