# Supplementary material for: A survey of NHS nurses' delivery of treatments to prevent recurrence of venous leg ulcers
Source: Int Wound J. 2025 Jan 12;22(1):e70101. doi: 10.1111/iwj.70101 (PMC11725358; doi:10.1111/iwj.70101)
Supplement: Supplementary file 1 — Data S1. Supporting information. [file IWJ-22-e70101-s003.docx]

**Supplementary file 1**

Questionnaire items (adapted; alternative text is presented in italics)

1. I have the PHYSICAL opportunity to *change my behaviour to improve my health*.

**What is PHYSICAL opportunity?**

The environment provides the opportunity to engage in the activity concerned.

(e.g. sufficient time, the necessary materials, reminders)

Please rate

Strongly Agree

Strongly disagree

0 1 2 3 4 5 6 7 8 9 10

☐ ☐ ☐ ☐ ☐ ☐ ☐ ☐  ☐ ☐

1. I have the SOCIAL opportunity *to change my behaviour to improve my health*.

**What is SOCIAL opportunity?**

Interpersonal influences, social cues and cultural norms provide the opportunity to engage in the activity concerned

(e.g., support from friends and family)

Please rate

Strongly Agree

Strongly disagree

0 1 2 3 4 5 6 7 8 9 10

☐ ☐ ☐ ☐ ☐ ☐ ☐ ☐  ☐ ☐

1. I am motivated to *change my behaviour to improve my health*.

**What is motivation?**

Conscious planning and evaluations (beliefs about what is good and bad)

(e.g. I have the desire to, I feel the need to)

Please rate

Strongly Agree

Strongly disagree

0 1 2 3 4 5 6 7 8 9 10

☐ ☐ ☐ ☐ ☐ ☐ ☐ ☐  ☐ ☐

1. *Changing my behaviour to improve my health* is something that I do automatically.

**Automatic motivation** involves doing something without thinking or having to consciously remember

(e.g. ‘is something I do before I realise I’m doing it’)

Please rate

Strongly Agree

Strongly disagree

0 1 2 3 4 5 6 7 8 9 10

☐ ☐ ☐ ☐ ☐ ☐ ☐ ☐  ☐ ☐

1. I am PHYSICALLY able to *change my behaviour to improve my health*.

**What is PHYSICAL capability?**

Having the physical skill, strength or stamina to engage in the activity concerned.

(e.g. I have sufficient physical stamina, I can overcome disability, I have sufficient physical skills)

Please rate

Strongly Agree

Strongly disagree

0 1 2 3 4 5 6 7 8 9 10

☐ ☐ ☐ ☐ ☐ ☐ ☐ ☐  ☐ ☐

1. I am PSYCHOLOGICALLY able to *change my behaviour to improve my health*.

**What is PSYCHOLOGICAL capability?**

Knowledge and/or psychological skills, strength or stamina to engage in the necessary thought processes for the activity concerned.

(e.g. having the knowledge, cognitive and interpersonal skills, having the ability to engage in appropriate memory, attention and decision making processes).

Strongly Agree

Please rate

Strongly disagree

0 1 2 3 4 5 6 7 8 9 10

☐ ☐ ☐ ☐ ☐ ☐ ☐ ☐  ☐ ☐
