# Supplementary material for: A survey of NHS nurses' delivery of treatments to prevent recurrence of venous leg ulcers
Source: Int Wound J. 2025 Jan 12;22(1):e70101. doi: 10.1111/iwj.70101 (PMC11725358; doi:10.1111/iwj.70101)
Supplement: Supplementary file 2 — Data S2. Supporting information. [file IWJ-22-e70101-s005.docx]

**Supplementary file 2**

**Survey Items**

**Section 1:**

**Demographic Information**

**Please completed the following demographic information as it applies to you**

**Gender (please select one response)**

Male Female Prefer not to say

**Age in years:**

**Ethnicity (please select one response):**

| White | Asian or Asian British | Black, Black British, Caribian or African | Mixed or Multiple ethnic group | Other ethnic group |
| --- | --- | --- | --- | --- |

**Information about your nursing role and where you deliver wound care**

| **What is your current nursing role (please selected one option):** | |  |  |  | |  |
| --- | --- | --- | --- | --- | --- | --- |
| Community nurse  Practice nurse | |  |  | | | |
|  | |  |  | | | |
| Tissue viability nurse | |  |  | | | |
| Other nurse role (please note). | |  |  | | | |
|  | |  |  | | | |
| **What is your current band (please select one response)?**   \| 2 \| 3 \| 4 \| 5 \| \| --- \| --- \| --- \| --- \| \| 6 \| 7 \| 8a \| Other please specify \| |  | | |  | |  |
| \| **For how long (to the nearest full year) have to delivered wound care in the NHS** \| \| \| \| \| \| \| --- \| --- \| --- \| --- \| --- \| --- \| \|  \|  \|  \|  \|  \|  \| | | | | |  | |
|  |  |  | | |  | |
|  |  |  | | |  | |
|  |  |  | | |  | |

| **Which NHS region do you work in?**   \| NHS England - North \|  \| \| --- \| --- \| \| NHS England -Midlands and East \|  \| \|  \|  \| \| NHS England-South- West \|  \| \| NHS England-South- East \|  \| \| NHS England-London \|  \| \| NHS Scotland \|  \| \| NHS Wales \|  \| \| NHS Northern Ireland \|  \|   **Service:**  **Where do you provide venous leg ulcer care? Please select all relevant options** |  |  |
| --- | --- | --- | --- | --- | --- | --- | --- | --- | --- | --- | --- | --- | --- | --- | --- | --- | --- | --- | --- | --- |
|  |  |  |

| GP Practice | |  |  |  |
| --- | --- | --- | --- | --- |
| Patient’s place of residence | |  |  |  |
| Community clinic/Treatment room | |  |  |  |
| Outpatient clinic | |  |  |  |
| In-patient settings | |  |  |  |
| Leg Club | |  |  |  |
| Hospice | |  |  |  |
| Other, please specify |  | | | |
|  |  | | | |

**Section 2:**

**Current venous leg ulcers prevention practices.**

**Please answer each of the questions below.**

**Provision of care:**

| Before today, were you aware of ‘the strongest level of compression hosiery tolerated’ as a treatment for people with healed venous leg ulceration to prevent recurrence? | | **Yes**  **No**  |  | |  |  |  |  |  |  |  |  |  |  |  |  |  |  |  |
| --- | --- | --- | --- | --- | --- | --- | --- | --- | --- | --- | --- | --- | --- | --- | --- | --- | --- | --- | --- |
| *If your answer is* ***NO,*** *please refer to the two links at the end of the survey for more information about venous leg ulcers preventive treatment*.  Of the people with healed venous leg ulcers you see in a typical working month, what proportion do you think would benefit from having the strongest level of compression hosiery they can tolerate to prevent recurrence? | | (Side bar; 0-100 %) |  | |  |  |  |  |  |  |  |  |  |  |  |  |  |  |  |
| Of people with healed venous leg ulcers you see in a typical working month who you think would benefit, with what proportion do you recommend use of the strongest level of compression hosiery tolerated to prevent recurrence?  When you offer compression hosiery to people with healed venous leg ulcers, what is the strongest level of compression you normally aim for?   \| Class 1   (14 to 17 mmHg) light support \| Class 2  (18 to 24 mmHg) Medium support \| Class 3  (25 to 40 mmHg) Strong support \| \| --- \| --- \| --- \| | | (Side bar; 0-100 %) |  | |  |  |  |  |  |  |  |  |  |  |  |  |  |  |  |
| Before today, were you aware of endo-venous ablation surgery in people with a history of venous leg ulceration as a treatment to prevent recurrence?  *If your answer is* ***NO,*** *please refer to the two links at the end of the survey for more information about venous leg ulcers preventive treatment*.  Of the people with healed venous leg ulcers you see in a typical working month, what proportion do you think would **benefit** from referral to vascular services to be considered for endo-venous surgery to prevent recurrence?  Of the people with healed venous leg ulcers you see in a typical working month who you think would benefit, what proportion do you **refer** to vascular services to be considered for endo-venous ablation to prevent recurrence? | | **Yes**  **No**         (Side bar; 0-100%)  (Side bar; 0-100%) | |  | | |  | | | | | |  | | |  |  |  |  |
| **Section 3**  **Please complete each question below.**  **What is PHYSICAL opportunity?**  The environment provides the opportunity to engage in the activity concerned. (e.g., sufficient time, the necessary materials, reminders) | |  |  | | | | | | | | | | |  | | |  |  |  |
| I have the PHYSICAL opportunity to recommend the strongest compression hosiery tolerated to people with a history of venous leg ulceration to prevent recurrence.  I have the PHYSICAL opportunity to refer people with a history of venous leg ulceration to vascular services to be considered for endo-venous ablation surgery to prevent recurrence  **What is SOCIAL opportunity?**  Interpersonal influences, social cues and cultural norms provide the opportunity to engage in the activity concerned. (e.g., other colleagues delivering compression for secondary prevention of venous leg ulcers, support from managers) | | **Please rate** Strongly 1 2 3 4 5 6 7 8 9 Strongly  Disagree agree 10  0                  0  **Please rate Strongly 1 2 3 4 5 6 7 8 9 Strongly**  **Disagree agree 10**  **0         **          0 |  | | | | | | | | | | |  | | |  |  |  |
| I have the SOCIAL opportunity to offer the strongest compression hosiery to people with a history of venous leg ulceration to prevent recurrence.  I have the SOCIAL opportunity to refer people with a history of venous leg ulceration to vascular services to be considered for endo-venous ablation surgery to prevent recurrence  **What is motivation?**  Conscious planning and evaluations (beliefs about what is good and bad) (e.g. I have the desire to, I feel the need to) | |  | **Please rate Strongly 1 2 3 4 5 6 7 8 9 Strongly**  **Disagree agree 10**  **0         **          0  **Please rate Strongly 1 2 3 4 5 6 7 8 9 Strongly**  **Disagree agree 10**  **0         **          0 | | | | | | | | | | |  | | |  |  |  |
|  |  | | | | | | | | | | | | |  | | |  |  |  |
| I am motivated to offer the strongest compression hosiery to people with a history of venous leg ulceration to prevent recurrence.  .  I am motivated to refer people with a history of venous leg ulceration to vascular services to be considered for endo-venous ablation surgery to prevent recurrence.  **Automatic motivation** involves doing something without thinking or having to consciously remember  (e.g. ‘It is something I do before I realise I am doing it’). | | **Please rate Strongly 1 2 3 4 5 6 7 8 9 Strongly**  **Disagree agree 10**  **0        **  ****          0  **Please rate Strongly 1 2 3 4 5 6 7 8 9 Strongly**  **Disagree agree 10**  **0        **           0 |  | |  |  | |  |  |  |  |  | | |  | | |  |  |
| Offering the strongest level of compression hosiery to people with a history of venous leg ulceration to prevent recurrence is something I do automatically.  Referring people with a history of venous leg ulceration to vascular services to be considered for endo-venous ablation surgery to prevent recurrence is something I do automatically.  **What is PHYSICAL capability?** Having the physical skill, strength or stamina to engage in the activity concerned. (e.g. I have sufficient physical stamina, I can overcome disability , I have sufficient physical skills)  (e. | | **Please rate Strongly 1 2 3 4 5 6 7 8 9 Strongly**  **Disagree agree 10**  **0       **            0  **Please rate Strongly 1 2 3 4 5 6 7 8 9 Strongly**  **Disagree agree 10**  **0       **            0 |  | |  |  | |  |  |  |  |  | | |  | | |  |  |
| I am PHYSICALLY able to offer the strongest level of compression hosiery to people with a history of venous leg ulceration to prevent recurrence  I am PHYSICALLY able to refer people with a history of venous leg ulceration to vascular services to be considered for endo-venous ablation surgery to prevent recurrence.  **What is PSYCHOLOGICAL capability?** Knowledge and/or psychological skills, strength or stamina to engage in the necessary thought processes for the activity concerned. (e.g. having the knowledge, cognitive and interpersonal skills, having the ability to engage in appropriate memory, attention and decision making process).  (e. | | **Please rate Strongly 1 2 3 4 5 6 7 8 9 Strongly**  **Disagree agree 10**  **0       **            0  **Please rate Strongly 1 2 3 4 5 6 7 8 9 Strongly**  **Disagree agree 10**  **0        **           0 |  | |  |  | |  |  |  |  |  | | |  | | |  |  |
| I am PSYCHOLOGICALLY able to offer the strongest level of compression hosiery to people with a history of venous leg ulceration to prevent recurrence  I am PSYCHOLOGICALLY able to refer people with a history of venous leg ulceration to vascular services to be considered for endo-venous ablation surgery to prevent recurrence  The two links below contain more information about the venous leg ulcers preventive treatment:  [Management of chronic venous leg ulcers. (SIGN Guideline No 120) (scot.nhs.uk)](https://www.nhstaysideadtc.scot.nhs.uk/wound%20Formulary/Pdf%20docs/sign120%202010.pdf) | | **Please rate Strongly 1 2 3 4 5 6 7 8 9 Strongly**  **Disagree agree 10**  **0   **          0  **Please rate Strongly 1 2 3 4 5 6 7 8 9 Strongly**  **Disagree agree 10**  **0**          0 |  | |  |  | |  |  |  |  |  | | |  | | |  |  |

[Lower Limb | National Wound Care Strategy Programme](https://www.nationalwoundcarestrategy.net/lower-limb/)
