# Supplementary material for: A survey of NHS nurses' delivery of treatments to prevent recurrence of venous leg ulcers
Source: Int Wound J. 2025 Jan 12;22(1):e70101. doi: 10.1111/iwj.70101 (PMC11725358; doi:10.1111/iwj.70101)
Supplement: Supplementary file 3 — Data S3. Supporting information. [file IWJ-22-e70101-s001.docx]

**Supplementary file 3**

**Six-step approach to Multivariate analysis**

Define the research problems, objectives and Multivariate technique to be used

Develop the analysis plan.

Evaluate the assumptions underlying the multivariate technique.

Estimate the multivariate model and assess overall model fit

Interpret the variables

Validate the multivariate model
