# Supplementary material for: A survey of NHS nurses' delivery of treatments to prevent recurrence of venous leg ulcers
Source: Int Wound J. 2025 Jan 12;22(1):e70101. doi: 10.1111/iwj.70101 (PMC11725358; doi:10.1111/iwj.70101)

Supplementary file 5

Four-step process for identifying missing data and reapplying remedies.

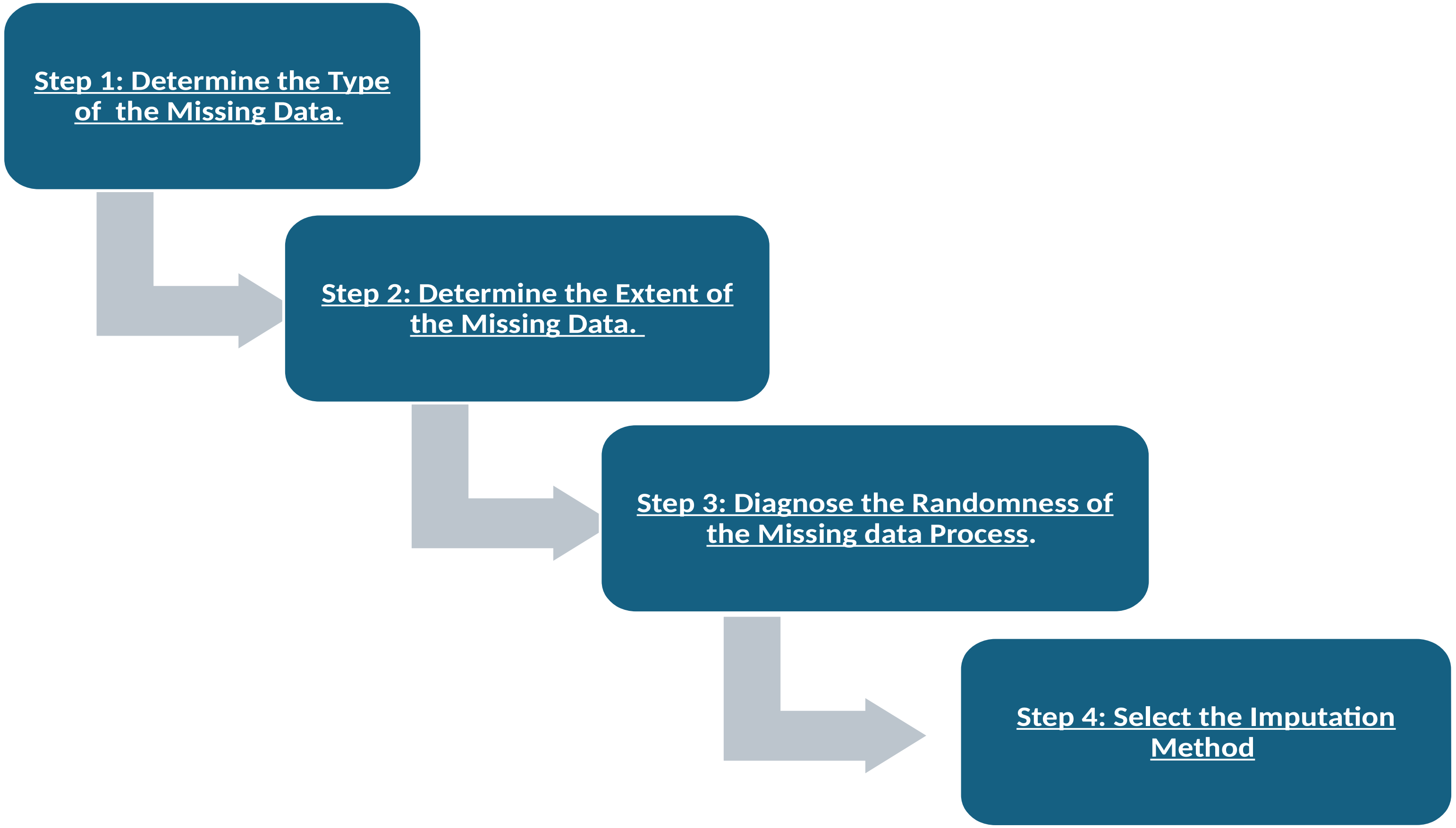

Supplement: Supplementary file 5 [file IWJ-22-e70101-s002.docx]
